# New Method of Producing Local Anæsthesia

**Published:** 1861-01

**Authors:** 


NEW METHOD OF PRODUCING LOCAL ANÆSTHESIA.—Mr. Richardson, the dentist, has sent us a communication, in which he says:—"The subject of local anæsthesia has much excited the attention of the profession lately. Dr. Richardson, Mr. Nunneley, and others, have devoted much time and research to it. Narcotics, congelation, inhalation of ether and chloroform, and, lastly, electricity, have all been tried, but from some practical disadvantages in their application, and from the occurrence of dangerous and even fatal effects none of these have met either the wants of the case or the general concurrence of the dental profession. In pursuing a series of experiments for effecting certain improvements in the 'Tooth Protector,' a notice of which apparatus was inserted in the *Lancet* of 1858, I have devised a plan for causing local anæsthesia during the extraction of teeth without producing a corresponding effect on the system generally—viz: by immersing the affected part in chloroform, and including in the part immersed as much of the adjacent structures as may be required. It is obvious that a plan so simple must be universally applicable, and should its reputation be maintained, prevent the escape of chloroform, and thus intensify its local effect. The cup is about half filled with cotton wool, which is then saturated with a sufficient quantity of chloroform, generally from ten to fifteen drops. The time within which local insensibility is produced varies from seven minutes to a quarter of an hour. It is true that the local anæsthesia is not, in all cases, equally complete; but even where pain occurs, the remedy will be found to moderate it to a point within which it becomes perfectly tolerable, and has lost the distressing agony of tooth extraction. It is important for the operator to remember, that as insensibility of the part ensues, the cup should be removed, and extraction instantly performed. Of sixty cases, on which the foregoing statements are based, only two occurred in which the remedy failed to mitigate the pain; whilst in ten cases the local insensibility during extraction was complete."—*Lancet.*

